# Large Conization—Retrospective Monocentric Results for Fertility Preservation in Young Women with Early Stage Cervical Cancer

**DOI:** 10.1007/s43032-021-00807-9

**Published:** 2021-11-29

**Authors:** Christos Tsaousidis, Bernhard Kraemer, Stefan Kommoss, Andreas Hartkopf, Sara Brucker, Katrin Neis, Juergen Andress, Felix Neis

**Affiliations:** 1 Women’s Center Bern, Lindenhofgruppe, Bern, Switzerland; 2 Department of Women’s Health, Women’s University Hospital, Tuebingen University Hospital, Calwerstr. 7, 72076 Tuebingen, Germany

**Keywords:** Large conization, Trachelectomy, Fertility preservation, Cervical cancer

## Abstract

The shorter cervical segment after classic radical trachelectomy (RT) imposes a number of pregnancy associated risk factors. In this aspect, large conization (LC) could be an oncologically safe alternative to RT in young women with early stage cervical cancer who want to spare their fertility. Our aim was to evaluate fertility-sparing surgical treatment of early stage cervical cancer after the introduction of LC. Our objectives were to assess surgical, oncological, fertility and obstetric outcomes. We retrospectively investigated oncological and fertility outcomes of patients who underwent LC in a large oncological single University centre between 2009 and 2014. Medical records were reviewed and analysed for surgical, oncological, fertility and obstetric outcomes. Postal questionnaires were collected to further evaluate and validate the fertility and obstetric outcomes. A total of 23 LCs were analysed. Seven patients had to undergo secondary radical hysterectomy after LC due to unclear resection margins. Nine of 16 women tried to conceive, of which all nine became pregnant. Seven patients underwent a prophylactic cerclage between 13 and 16 gestational weeks and seven women delivered 9 children; the majority of women conceived spontaneously. Follow-up time was a median of 3.9 years (2.6–8 years). There was no relapse of cervical cancer in the investigated timeframe. Early stage cervical cancers treated by LC are associated with excellent oncological outcomes. LC appears to be a safe option for eligible women who intend to maintain their fertility.

## Introduction

Cervical cancer is the fourth most common cancer in women, and the tenth overall, with 569,847 new cases in 2018 worldwide [1]. Increasingly, young women with no children are affected. In Germany, 36 out of 100,000 women between the ages of 20 and 40 are diagnosed with cervical cancer every year [2]. Due to social and economic reasons, family planning shifts towards the end of the third decade and into the fourth decade of a woman’s life.

Depending on the stage of the disease, cervical cancer is traditionally treated by (radical) hysterectomy (RH) and pelvic lymph node dissection (PLND). Fertility-sparing therapy options are important to young women in their reproductive life if their oncological safety is considered. Besides the psychological and physical distress after cancer diagnosis, the ability to conceive during their reproductive period is an issue for patients with excellent prognosis. Therefore, it is important to offer women with early cervical cancer (tumour size ≤ 2 cm, no lymphovascular space invasion, no lymph node involvement) who wish to conceive a treatment that preserves fertility [3].

The first successful systematic conservative surgical approach for invasive cervical carcinoma was described and published by Dargent in 1994 [4, 5]. This procedure included laparoscopic pelvic lymphadenectomy and vaginal radical trachelectomy (VRT) also referred to as the ‘Dargent operation’.

The classical radical trachelectomy basically consists of the resection of a cervical segment including parametrial tissue after the mobilisation of the ureter, the sparing of the uterine artery and the resection of vaginal tissue. The characteristic feature of a less radical modification (simple vaginal trachelectomy, SVR) is usually the lack of any relevant ureter dissection. To date, there are no definite recommendations on the use of these different techniques.

Since their introduction, trachelectomy procedures are using both abdominal and vaginal approaches with different margins and surgical routes such as conventional and robotic-assisted laparoscopy, vaginal and abdominal surgeries and various combinations [6–9]. Trachelectomy combined with PLND is currently considered an option in women with early stage cervical cancer and a desire for future reproduction [7, 10].

Although RT is suggested as a fertility preserving approach, there are some serious side effects which affect the future integrity and function of the remaining uterine architecture. Gestation associated side effects are especially second trimester loss, premature rupture of the membranes (PROM) due to chorioamnionitis and preterm delivery. Another very important information which needs to be discussed with the patient before surgery is the fact, that after LC or RT no vital tumour cells are found in the majority of the removed specimen and that there is a risk of postoperative cervical stenosis [11–13].

Preliminary findings of less radical procedures (deep cone and simple trachelectomy) in patients with tumours less than 2 cm, and negative sentinel and other pelvic lymph nodes, are demonstrated to be comparable with the results of RT [14]. As a consequence, these less extensive surgical options should be further evaluated. Several national and international guidelines describe LC as a treatment option in women with early cervical cancer and desire for fertility preservation [15, 16].

In this aspect, our aim was to analyse LC in early stage cervical cancer in a single centre university hospital setting regarding oncological, fertility and obstetric outcomes.

## Material and Methods

### Study Design

After the approval of the ethical committee of the university (No. 027/2015BO2), a retrospective analysis of patients who underwent LC in our department was conducted (Clin Trial No. DRKS00012380). The research was carried out according to the principles set out in the Declaration of Helsinki 1964. All procedures were performed in accordance with the ethical standards of the institutional research committee.

Relevant clinical information regarding oncological and pregnancy outcome associated issues were collected from medical records and by means of a questionnaire that was sent out to all eligible women who underwent LC in our institution during a period of 6 years between January 2009 and December 2014.

Inclusion criteria for a LC were clinical tumour size < 2 cm (≤ FIGO stage Ib1, according to the FIGO classification of 2010), no evidence of lymphangiosis carcinomatosa (L0), no evidence of distant metastases and the patient’s current or prospective desire to preserve fertility, independent of current or prospective desire to have children.

For preoperative staging, a CT scan of the thorax, abdomen and pelvis was performed. In unclear cases, an MRI of the pelvis was also conducted.

The classification and staging of cervical cancer were adapted to the FIGO staging system of 2018 [17].

### Postal Questionnaires

The postal questionnaires were sent to the patients who received LC at our institution within the time interval from 2009 and 2014. The questionnaire consisted of two parts. The first part focused on the clinical oncological follow-up. The second part focused on fertility issues (natural conception versus assisted reproduction) and obstetrical outcomes (preterm labour, PROM, time and mode of delivery).

The postal questionnaire included an informed consent letter, which was sent back to our institution by the patient together with the questionnaire. After receiving the written informed consent and questionnaire, the patient was included into this study.

### Surgical Technique of LC

**Fig. 1:**
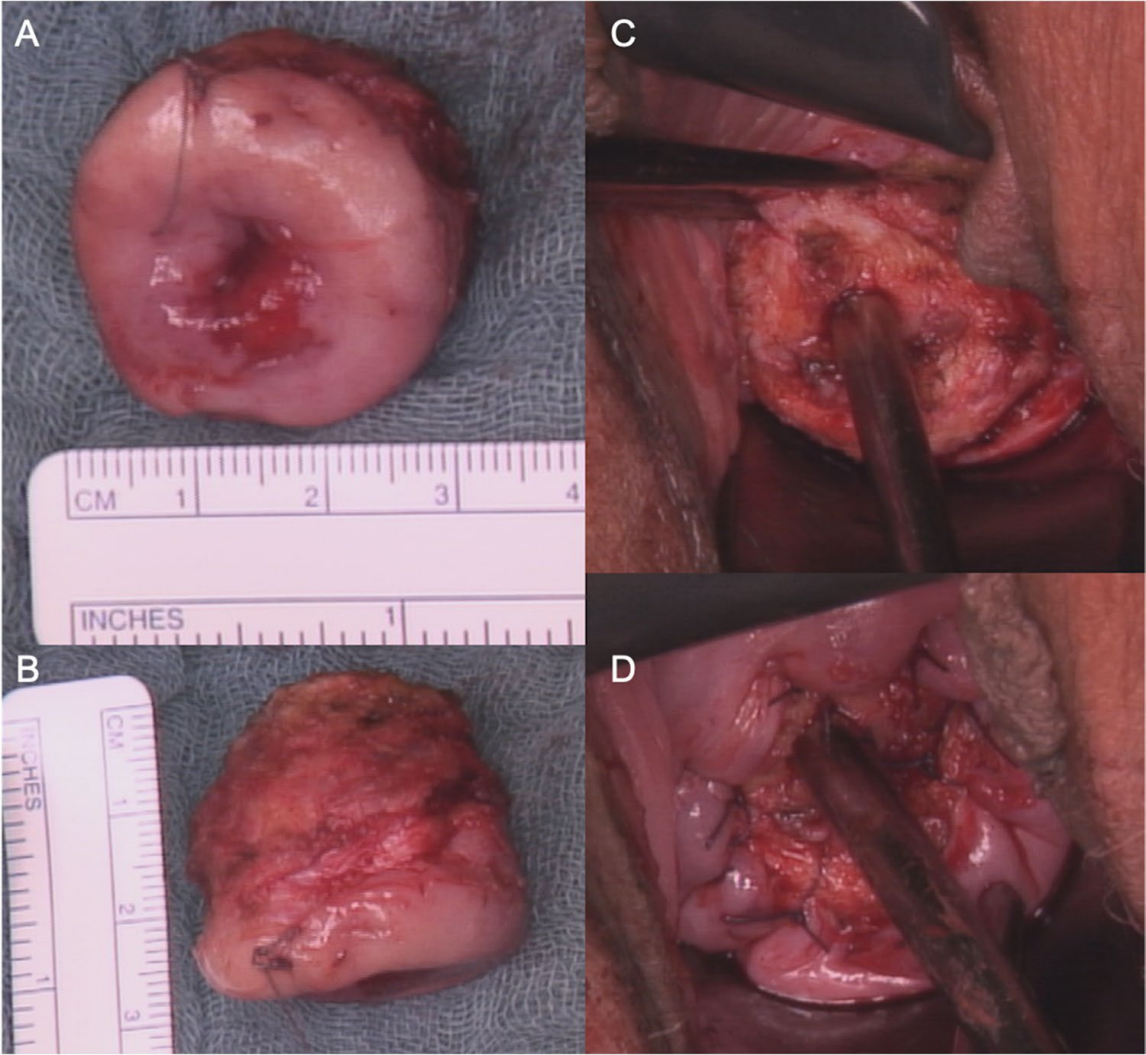
Patient with residual tumour after conization in the quadrant from 3 to 6 o’clock. LC specimen with scale (cm and inches): (**A**) view of the cervical canal, (**B**) view from the side. And postoperative situs without (**C**) and with sutures (**D**)

After general anaesthesia, the patients are positioned in lithotomy position. After ligation of the descendent branch of the uterine artery on both sides at 3 and 9 o’clock position, the cervical uterine segment is pulled down towards the introitus for optimal exposure. A circular incision of the vaginal wall is performed with a monopolar needle. Without opening the peritoneal cavity, the vaginovescial, the rectovaginal and the lateral columns are largely mobilised from the cervical wall in order to expose the tumour region and the cervix up to the cervicouterine level. The key moment is the cervicectomy with a curved monopolar needle including the tumour region and a macroscopically clear cervical margin of 1–2 cm without the transection of parametrial or paracolpium tissue. Haemostasis is achieved by electrocoagulation and the attachment of the vaginal cuff to the remaining cervical stump with ≥ 8 Vicryl single knot sutures of which four are placed at 12, 3, 6 and 9 o’clock position and at least four more are positioned between each of the previous ones. The removed specimen and the postoperative situs are demonstrated in Fig. 1.

After conception, a prophylactic cerclage according to McDonald [18] was recommended between 13 and 16 gestational weeks.

#### Histopathological Workup: Resection Margins and Lymph Nodes

After large conization, an extensive histopathological examination was performed by a specialised gynecologic pathologist. The conization specimen was analysed for type of cancer, grading, lymphovascular involvement and margins of the tumour according to the current WHO/IARC classification.

The margins were defined as R1 if a tumour cell was visible at the margin of the conus. If there was healthy tissue of more than 3 mm, the resection status was defined as R0.

This leads to two possible scenarios:

- In case of clear margins: a subsequent laparoscopic pelvic lymphadenectomy combined with sentinel lymph node dissection (Tc-99 m-SLND) is performed, if indicated, to complete staging within 1 week after LC.
- In case of R1 resection: an extrafascial or (modified) radical laparoscopic hysterectomy according to tumour stage including pelvic lymphadenectomy combined with Tc-99 m-SLND is performed to complete staging within 1 week after LC.

In case of lymphonodectomy, the sentinel lymph nodes were examined by ultrastaging and the other lymph nodes by routine histopathological analysis.

### Statistical Analyses

Due to the small size of the retrospective study cohort, this investigation is focused on descriptive and basic statistical evaluation (Excel® system and SPSS®).

## Results

### Baseline Characteristics of the Patients

Table 1 depicts the baseline characteristics of the retrospective study cohort. During the investigation period, a total of 23 women underwent LC who were eligible for inclusion. The mean patient age was 31.4 years, the mean BMI was 23.2 kg/m^2^. In 16 women, LC successfully led to the complete removal of cervical cancer indicated by the pathological report as clear margins (R0 resection). Nearly one-third (n = 7, 30.4%) had to undergo secondary radical hysterectomy due to tumour size and involvement of the margins in the final LC specimen. These patients were excluded from the follow-up time.

**Table 1:** Patients characteristics (BMI, body mass index; LC, large conization)

Characteristics	*n* = 23	
Age (years)		
Median (range)	32 (25–37)	
BMI (kg/m^2^)		
Median (range)	23.2 (18.8–38.1)	
FIGO stage (*n* (%))	Classification of 2010	Classification of 2018
1a1	6 (26.1)	5 (21.7)
1a2	4 (17.4)	4 (17.4)
1b1	13 (56.5)	8 (34.8)
1b2	0 (0)	6 (26.1)
Maximal tumour size after final surgery (mm (range))		
Median tumour size (mm (range))	9 (2–38)	
Median tumour size if after LC R0 (mm (range))	7 (2–32)	
Median tumour size if after LC R1 (mm (range))	32 (10–38)	
Depth of infiltration after final surgery (mm (range))		
Median depth of infiltration if after LC R0 (mm (range))	3.6 (1–10)	
Median depth of infiltration if after LC R1 (mm (range))	12 (5–34)	
Histological type, *n* (%)		
Adenocarcinoma	13 (56.5)	
Squamous	10 (43.5)	
Grade, *n* (%)		
1	2 (8.7)	
2	14 (60.9)	
3	7 (30.4)	
Pelvic node count		
Median (range)	21.5 (3 – 39)	
Deep of LC (mm (range))		
Deep of LC if R0, median (mm (range))	22 (11–37)	
Deep of LC if R1, median (mm (range))	33 (15–50)	

Fifteen patients (65.2%) underwent a conization prior to LC without previous biopsy due to a pap smear suspecting HSIL, the other eight patients (34.8%) had a colposcopically guided biopsy without conization before LC. 43.5% (10/23) were diagnosed with squamous cell carcinoma and 56.5% (13/23) of the patients presented with adenocarcinoma. In the adenocarcinoma group, one patient has an adenosquamous carcinoma of the cervix. As the highly dominant component is an ACC, this patient was assigned to the ACC group. All patients had a clinical tumour size according to FIGO Ib1 (2018) or smaller. After final histological examination, 17 patients had a tumour size ≤ 2 cm. The average diameter of all tumours was 9 mm (2–38 mm). If there were clear margins in the final histological examination, the median size was 7 mm (range 2–32 mm); in case of a R1-resection, the median was 32 mm (10–38 mm). For all patients with a R0-resection, the tumour was more than 4 mm away from the resection margin. 69.6% (16/23) were classified as grades 1 or 2, approximately one-third of our patients had grade 3 carcinomas.

All patients who had an indication for pelvic lymphadenectomy (20/23, 87%) had no tumour involvement of pelvic lymph nodes. The 3 patients without indication for pelvic lymphadenectomy had microinvasive cancer without risk factors and were tumour stage FIGO 1a1, G1-2 and L0.

If a LC presented clear margins, the depth of the cone was 22 mm (range 11–37 mm); in cases of involvement, the cone was 33 mm (range 15–50 mm). In all 7 patients with involved margins, a radical hysterectomy procedure was performed after LC.

If there were clear margins within the LC, the infiltration depth was 3.6 mm (range 1–10 mm); if the margins were involved, the median infiltration depth was 12 mm (range 5–34 mm) (Table 2). In the case of affected margins, tumour grading was G3 in the majority (57.1%), if the margins were free of tumour only 18.75% were G3.

**Table 2:** Comparison of tumours when large conization R0 and R1 (LC, large conization)

		LC R0, ***n*** = 16	LC R1, ***n*** = 7
Histological type of tumour	Adenocarcinoma (*n*, %)	9 (56.25%)	4 (57.1%)
	Squamous (*n*, %)	7 (43.75%)	3 (42.9%)
Grading	G1	2 (12.5%)	0 (0%)
	G2	11 (68.75%)	3 (42.9%)
	G3	3 (18.75%)	4 (57.1%)
Size	Median size of tumour after final histological examination (mm (range))	7 (2–32)	32 (10–38)
	Deep of LC Median (mm (range))	22 (11–37)	33 (15–50)
	Median depth of infiltration if after LC (mm (range))	3.6 (1–10)	12 (5–34)
Lymphovascular involvement	L1 (*n*, %)	3 (18.75%)	1 (14.3%)

### Follow-up Time

The follow-up time was monitored both by the retrospective analysis of patient’s hospital records and a postal questionnaire that was sent out to the patients in order to gain information about obstetrical outcomes after cancer treatment, especially in cases when patients had left the catchment area of our hospital. The follow-up time was between 31 and 96 months (Table 3).

**Table 3:** Follow-up if large conization was successful

All	*n* = 16
Follow-up time (months): median (range)	58 (31–96)
Disease recurrence, *n* (%)	0 (0)
Regular menstruation, *n* (%)	13 (81.3)
Secondary hysterectomy, *n* (%)	2 (12.5)

### Oncologic Outcomes

During follow-up time, none of the LC patients had a disease recurrence and all of the follow-up controls (clinical investigation and pap smears) were without any suspicious or pathological findings based on the data that were returned in the questionnaire for analysis.

With regard to abnormal uterine bleedings, three LC patients (3/16, 18.7%) reported about an irregular menstrual bleeding after the surgery.

### Fertility and Obstetric Outcomes

Nine of 16 women tried to conceive during the investigation period, of which all nine became pregnant. Seven patients had nine live births. During all pregnancies, a cervical cerclage between thirteen and sixteen gestational weeks was necessary. Four patients (28.6%) had a spontaneous miscarriage before the 15th week of gestation and one patient had an extrauterine pregnancy. Spontaneous conception occurred in 71.4% (10/16). In 4 cases (4/14, 28.6%), assisted reproduction techniques were used. Table 4 shows the fertility outcome after LC.

**Table 4:** Fertility outcome of the patients after a large conization (LC, large conization; IVF, in vitro fertilisation; EUG, extrauterine gestation; PROM, prelabor rupture of membranes)

All	*n* = 9
Patients who became pregnant, *n* (%)	9 (100)
Patients who had a childbirth, *n* (%)	7 (77.8)
No. of pregnancies, *n*	14
Childbirths, *n* (%)	9 (64.3)
Spontaneously miscarriages < 15 weeks, *n* (%)	4 (28.6)
EUG, *n* (%)	1 (7.1)
Conception, *n* (%)	
Spontaneously	10 (71.4)
IVF	4 (28.6)
Cerclage, *n*	9
Cerclage week, median (range)	15.1 (13–16)
Delivery week	36 (28–39)
24 + 0–33 + 6	2
34 + 0–36 + 6	1
> 37 + 0	6
Art of delivery, *n* (%)	
Vaginal delivery	1 (11)
Caesarean section	8 (89)
Complications	
PROM, *n* (week)	2 (26 and 28)

In this study, a couple of young patients with a desire to spare fertility, due to a prospective desire to have children were included. At the time of the postal questionnaire, however, the desire for pregnancy had not yet been actively pursued in some patients.

After delivery, two of the patients who conceived underwent a secondary hysterectomy as their family planning was completed and due to their desire for future oncological safety. Both of them had a benign histology without any signs of suspicion in the remaining cervical tissue after LC.

## Discussion

Fertility preservation is becoming a major issue for certain types of cancer in gynaecological oncology due to the fact that there is a shift to complete family planning in later years of a women’s lifetime with postponement of childbearing. This coincides with the morbidity rate of cervical cancer. In this aspect, fertility-sparing surgery has become an option for young patients with early stage cervical cancer [14, 19], and radical trachelectomy (RT) is widely accepted in the literature for that purpose.

Cervical stenosis after RT and related complications (for example menstrual disorders) [20, 21] are seen as relevant side effects. Another important concern is the lack of residual tumours in 60% in the specimens after RT which could be interpreted as a potential overtreatment [11, 22].

Consequently, an alternative surgical treatment without compromising oncological safety but improved pregnancy outcomes is under investigation of which simple vaginal trachelectomy (SVT) and LC could be options with promising results. However, it has to be stated that to our knowledge to date, there is no exact definition of radical versus simple trachelectomy as well as large or deep conization. In addition, both the pattern of recurrence and recurrence rates after conization and pelvic lymphadenectomy in patients with early stage cervical cancer remain unclear [11, 22].

However, traditional RT traumatises and subsequently weakens the lower segment of the uterus, due to resection of the parametrium and removal of normal cervical stroma beyond the tumour. Therefore, the risk of second trimester loss and preterm delivery has been reported to be major complications during a pregnancy after RT [21]. In a systematic literature review on RT, 413 out of 485 patients (85%) were able to maintain their fertility. A total of 113 patients (38%) attempted to get pregnant, and 67 of them (59.3%) were able to conceive [20]. In our study, although the number of patients is less, nine of sixteen women intended to get pregnant (56.2%) and all 9 of them (100%) were able to conceive. There were 4 miscarriages (28.6%), which is in a similar range as described in recent literature [20, 23, 24]. Despite the rate of spontaneous conceptions was high (71.4%) in our study, 4 patients needed assisted reproductive technology (ART).

The rate of patients who delivered after LC was 64% which is comparable to previous studies [10, 13], highlighting a promising pregnancy rate after LC. In our cohort, 77.8% of the children were born after gestational week (gw) 34 + 0 and 66.7% were born after gw 37 + 0, which is comparable to the results of Plante et al. [10]. However, a recent review stated that the risk of prematurity probably varies according to the surgical approach, ranging from 39 to 57%, with a significantly higher rate of prematurity after abdominal RT [13]. To date, 21 RT are described during pregnancy in early stage cervical cancer, showing the feasibility and safety even in high-risk situations such as in pregnant women [25]. According to the depth of LC, the risk of preterm delivery might rise. In the group with successful LC, the depth of the removed specimen was 21.7 mm on average ranging from 11 to 37 mm. Literature describes the anatomical length of the cervix uteri around 3 ± 1 cm [26–28]. Castanon could show in his analysis of patients undergoing conization a doubled risk of preterm delivery if the depth of resection was larger than 1.5 cm and an OR of even 2.4 if the resection was deeper than 2 cm [29]. A study by Simoenes characterises the risk of preterm delivery after resection of more than 1 cm with an OR of 4.55 [30]. A benefit of cervical cerclage in case of a cervical length of 15 mm could be shown by Owen [31]. To reduce the risk of preterm delivery, all pregnant patients received a cerclage in our cohort.

Our study shows an acceptable level of successful LCs with strict oncologic criteria, and satisfactory oncological safety, fertility and obstetric outcomes.

The oncological safety of LC is comparable to patients after RT. None of our patients had a recurrence. A recent meta-analysis showed an altogether low recurrence rate of 2.3% after RT, ranging from 0 to 33% in analysed studies [32]. However, the follow-up times are different in the published studies and our study.

Tseng et al. compared less radical surgeries (LC, TR, simple hysterectomy) and radical surgeries (modified radical and radical hysterectomies) in patients with stage IB1 (classification of 2009) cervical cancer. They demonstrated that there was no difference in disease-specific survival (DSS) after 10 years of follow-up in these cancer stages. As independent risk factors for increased risk of recurrence, they found adenosquamous histology, tumour grade 3, tumour size larger than 2 cm and lymph node metastasis [33]. Our cohort consists mainly of tumour sizes smaller than 2 cm, 69.6% G1-2, and we had no lymph node involvement in our patients. This might explain the low rate of recurrence.

Of great importance is the extent of the tumour and the negative lymph node status [3, 15]. Cai et al. analysed the frequency of lymph node involvement in 289 patients with FIGO stage IB1 (classification of 2009) and 8314 lymph nodes [34]. They showed an incidence of 15.22% of lymph node metastasis in this specific group. A tumour size of more than 2 cm, histologically proven lymphovascular space involvement and parametrial invasion were significantly correlated with a higher risk of lymphatic metastasis [34]. In our cohort, we performed a three-step lymph node exploration. Preoperatively, all patients received a CT scan and in unclear situations an MRI scan. According to the personal risk of the patient, a sentinel-guided pelvic lymph node dissection was performed. Only in case of a microinvasive cancer without risk factors, tumour stage FIGO 1a1, G1-2 and L0 surgical lymph node assessment was omitted. Histological ultra-staging was used for lymph nodes, routine histopathological analysis for the other lymph nodes. We did not find any involved lymph nodes in our study. The first reason for this might be the small sample size of our cohort. The second reason might be the strict selection criteria we used for LC, as the median size of the tumours was 7 mm with a range of 2 mm to a maximum of 32 mm and only 18.75% had a lymphovascular involvement.

In the presented study, patients with clinical tumour size smaller than 2 cm were included. However, the final histologic findings revealed six women with significantly larger tumours. In the group of patients with R1 resection, five of seven patients had tumours larger than 2 cm. In the group of successful LC, only one patient had a tumour larger than 2 cm. This demonstrates the difficulty of correctly clinically assessing tumour size as a requirement for LC. Therefore, the preoperative clinical examination should be performed by an experienced gynaecological oncologist. The preoperative evaluation of the lymph nodes seems to be far more reliable. Thus, there were no lymph node metastases in the study population.

Comparing the patients with successful LC and those with the need for the secondary radical hysterectomy procedure, there was no difference regarding the histological type, which seems to play a minor role. However, the differences become apparent when tumour size and grading are considered. The final histologic result showed considerably larger tumours in the group with an R1 resection after LC. Affected margins were found about 3 times more frequently in tumours with a G3 status. Thus, grading appears to be an important prognostic factor for successful LC. In case of G3 carcinoma, the patient should be informed about the higher risk of a R1 situation before surgery.

Although 56.4% of our patients had an adenocarcinoma, the ovarian preservation seems to be no risk at early stage cervical carcinoma. A meta-analysis in 1427 patients with adenocarcinoma or squamous carcinoma of the cervix FIGO stage (CIS—IIA) who underwent hysterectomy, showed no ovarian recurrences after unilateral or bilateral ovarian preservation in adenocarcinoma patients in the follow-up (30–68 months); however, 15 patients with squamous carcinoma developed pelvic recurrence [35].

The interpretation of our study results is limited by a relatively small patient number and a nonrandomized setting. Secondly, there were six different oncological surgeons who performed the LC. This methodical problem was also shown by Shim et al. in a recent systematic review. They demonstrate the problem of analysing recurrence and pregnancy rates in early stage cervical cancer (IA1) with lymphovascular involvement treated by LC or SVT, since most studies were retrospective and of small, nonrandomized numbers [24]. In this respect, our results may contribute additional clinical data to further evaluate LC as a surgical technique that supports fertility preservation with less cervical trauma but with uncompromised oncological safety.

## Conclusion

LC is a surgical technique for the therapy of early stage cervical cancer in young women. The oncological safety is comparable to the results of studies with patients who underwent radical trachelectomy but with an improved fertility outcome. Despite that fact that none of the study patients had recurrent disease and all of the women who had the desire to conceive became pregnant during follow-up time, future prospective clinical trials with larger sample sizes are needed to confirm the positive findings. According to available data and our present results, we conclude for the daily practice that large conization can be offered to women with early stage cervical cancer after counselling and involvement in the decision-making process.

## Data Availability

All data generated or analysed during this study are included in this published article.

## Abbreviations

ART: Assisted reproductive technology
BMI: Body mass index
EUG: Extra uterine gravidity
GW: Gestational week
IVF: In vitro fertilisation
LC: Large conization
PLND: Pelvic lymph node dissection
PROM: Premature rupture of the membranes
RH: Radical hysterectomy
RT: Radical trachelectomy
SLND: Sentinel lymph node dissection
SVT: Simple vaginal trachelectomy
VRT: Vaginal radical trachelectomy
